# Genome-wide survey and analysis of microsatellites in giant panda (*Ailuropoda melanoleuca*), with a focus on the applications of a novel microsatellite marker system

**DOI:** 10.1186/s12864-015-1268-z

**Published:** 2015-02-07

**Authors:** Jie Huang, Yu-Zhi Li, Lian-Ming Du, Bo Yang, Fu-Jun Shen, He-Min Zhang, Zhi-He Zhang, Xiu-Yue Zhang, Bi-Song Yue

**Affiliations:** Key Laboratory of Bio-resources and Eco-environment (Ministry of Education), College of Life Sciences, Sichuan University, Chengdu, 610064 P R China; Sichuan Key Laboratory of Conservation Biology on Endangered Wildlife, College of Life Sciences, Sichuan University, Chengdu, 610064 P R China; Pharmacy College, Chengdu University of Traditional Chinese Medicine, Chengdu, 610075 Sichuan China; Chengdu Research Base of Giant Panda Breeding, Chengdu, 610081 China; China Research and Conservation Centre for the Giant Panda, Wenchuan, 623006 China

**Keywords:** *Ailuropoda melanoleuca*, Genome sequence, Tetranucleotide microsatellite, Marker system

## Abstract

**Background:**

The giant panda (*Ailuropoda melanoleuca*) is a critically endangered species endemic to China. Microsatellites have been preferred as the most popular molecular markers and proven effective in estimating population size, paternity test, genetic diversity for the critically endangered species. The availability of the giant panda complete genome sequences provided the opportunity to carry out genome-wide scans for all types of microsatellites markers, which now opens the way for the analysis and development of microsatellites in giant panda.

**Results:**

By screening the whole genome sequence of giant panda *in silico* mining, we identified microsatellites in the genome of giant panda and analyzed their frequency and distribution in different genomic regions. Based on our search criteria, a repertoire of 855,058 SSRs was detected, with mono-nucleotides being the most abundant. SSRs were found in all genomic regions and were more abundant in non-coding regions than coding regions. A total of 160 primer pairs were designed to screen for polymorphic microsatellites using the selected tetranucleotide microsatellite sequences. The 51 novel polymorphic tetranucleotide microsatellite loci were discovered based on genotyping blood DNA from 22 captive giant pandas in this study. Finally, a total of 15 markers, which showed good polymorphism, stability, and repetition in faecal samples, were used to establish the novel microsatellite marker system for giant panda. Meanwhile, a genotyping database for Chengdu captive giant pandas (n = 57) were set up using this standardized system. What’s more, a universal individual identification method was established and the genetic diversity were analysed in this study as the applications of this marker system.

**Conclusion:**

The microsatellite abundance and diversity were characterized in giant panda genomes. A total of 154,677 tetranucleotide microsatellites were identified and 15 of them were discovered as the polymorphic and stable loci. The individual identification method and the genetic diversity analysis method in this study provided adequate material for the future study of giant panda.

**Electronic supplementary material:**

The online version of this article (doi:10.1186/s12864-015-1268-z) contains supplementary material, which is available to authorized users.

## Background

The giant panda (*Ailuropoda melanoleuca*), a global icon of biodiversity conservation, is threatened by human population expansion and current habitat loss and is often cited as one of the most endangered species in the world [1,2]. The international society and the Chinese government have made great efforts to protect this precious species in recent years. However, some urgent problems are still left unsolved.

Currently, the conservation strategy for the giant panda includes both captive pandas and wild pandas. Until 2013, the captive population size had grown to 376 individuals, more than 200 of which were living in Chengdu Research Base of Giant Panda Breeding (Chengdu, China) and China Research and Conservation Center for the Giant Panda (Wolong, China). The paternity of panda offspring bred in captivity is uncertain due to the breeding pattern in which a female in estrous is artificially inseminated with the sperm from multiple males. As a result, it has been impossible to maintain an accurate studbook; therefore, an accurate paternity assignment method needs to be established for the captive population. In addition, the design of conservation strategies for the wild panda population is also limited by the lack of information on the population’s genetics. Although microsatellite loci analyses [3-8] have been used to assess the genetic variability and evaluate the population size for giant pandas, the genetic status of the giant panda is still matters of significant controversy. For example, some researchers assumed that wild populations might have low genetic variability [9-11], while Lu et al. [3] and Zhang et al. [5] concluded that wild populations might maintain high genetic variation. However, it is difficult to make comparisons between the different results due to the different microsatellites they use, which is really confused the conservator in designing effective conservation strategies. Therefore, a universal genetic marker system, which is powerful and repeatability, will be convenient for different researchers to make comparison. Although nearly 100 microsatellite markers have already been developed for the giant panda [3,8,12-16], most of them are dinucleotide repeats. Dinucleotide microsatellite is easily subject to mistyping due to polymerase slippage during polymerase chain reaction (PCR) [17,18]. This problem is especially acute when template DNA is of low quality or concentration, as with faecal samples or degraded tissue samples [9,19,20]. The high quality samples are very difficult to obtain from wild. Schlotterer and Tautz [21] also found the generation of false alleles from polymerase slippage is greatest with di-, less with tri-, and does not occur with tetranucleotide loci. In general, tetranucleotide repeats tend to stutter less than the trinucleotide and dinucleotide repeats and are much more accurate and reliable [22,23], which also has become the marker of preferred choice and be widely used in paternity test kits for people [24,25]. Disappointingly, only 15 markers with single motif of (GATA)n were tetranucleotide repeats and nearly no one were used in the wild genetic studies. It was because most of them were unavailable when using the non-invasive samples. In this study, we focused on developing microsatellites with high levels of polymorphism, strong stability, good repeatability, and very low genotyping error rate, which would be widely used in the giant panda studies. Therefore, we concentrated on the tetranucleotide microsatellites to establish a universal genetic marker system.

Classically, microsatellite development requires substantial technical effort to construct enriched microsatellite libraries, including cloning, hybridization to detect positive clones, plasmid isolation, and Sanger sequencing [26]. Most of these steps are either expensive, time-consuming, or both. Moreover, traditional enrichment-based approaches for isolating microsatellite loci require *a priori* choices about what types of microsatellite loci to target (both repeat size, and repeat motif sequence) which will ultimately lead to limited success in obtaining sufficient numbers of different types of useful microsatellite loci [26]. Fortunately, the availability of the giant panda complete genome sequences [27] provided the opportunity to carry out genome-wide scans for all types of microsatellites markers, which is much cheaper, more efficient and more successful than the previous methods. Consequently, a diversity of repeat motif types of microsatellites can be identified so as to establish a universal genetic marker system for giant panda.

Here we employ a method that allows the rapid and efficient development of microsatellite markers for giant panda by screening its whole genome sequence *in silico* mining. A large number of different kinds of repeat motif types of perfect microsatellite sequences were discovered. Moreover, the frequency and distribution of these microsatellites in different genomic regions were analyzed and an integrative database of tetranucleotide microsatellite markers was developed. The 51 novel polymorphic tetranucleotide microsatellite loci screened from the database were further used to establish the universal genetic marker system for giant pandas with faecal samples. Furthermore, a universal individual identification method was established, which is particularly effective in assessing the population size for wild giant pandas. We also analyzed the genetic diversity of Chengdu captive giant panda population.

## Results

### SSR frequency and distribution in the giant panda genome

A total of 855,058 SSRs were identified in the giant panda genome assembly (Table 1). The relative abundance was 372 SSRs/Mb. Mono-nucleotides were the most abundant category, accounting for 48.56% of all of the SSRs, followed by di-nucleotides (26.17%) and tetra-nucleotides (18.09%). In contrast, tri-nucleotides and penta-nucleotides were less abundant.

**Table 1:** **Distribution of microsatellite with respect to motif length in the giant panda genome**

**Motif length (bp)**	**Mono-**	**Di-**	**Tri-**	**Tetra-**	**Pentra-**	**Hexa-**	**Total**
No.	415,195	223,765	35,917	154,677	23,167	2,337	855,058
Length (bp)	6,166,765	4,223,706	636,042	3,206,684	625,100	625,100	61,692
Abundance(No./Mbp)	180.54	97.3	15.62	67.26	10.07	1.02	371.81
Percent of each repeat(%^a^)	48.56%	26.17%	4.20%	18.09%	2.71%	0.3%	

%^a^ = no./total no. of microsatellites.

Among all the mono-nucleotide repeats, (A)n was the most abundant while (C)n was comparatively scarce (Table 2). In the di-nucleotide repeat category, (AC)n and (AG)n were the two most frequent microsatellite motifs. Over 50% of the trinucleotide type were (AAC)n and (AAT)n in the panda genome. The most abundant tetra- and penta-nucleotide motifs were (AAAT)n and (AAACA)n, which comprised about 42.03% and 31.76% of the total number of microsatellites of these two repeat category, respectively. (AAACAA)n was the most frequent hexa-nucleotide motifs. A-rich occurred in nearly all the most frequent motifs of microsatellites.

**Table 2:** **The most frequent microsatellite motifs found in the giant panda genome sequences**

	**Repeat motifs**					
	**Mono-**	**Di-**	**Tri-**	**Tetra-**	**Penta-**	**Hexa-**
	A(95.41)	AC(45.27)	AAT(29.77)	AAAT(42.03)	AAACA(31.76)	AAACAA(21.44)
*A.melanoleuca*	C(4.59)	AG(44.48)	AAC(29.19)	AAAG(16.73)	AAAGA(20.71)	AAAGAA(9.5)
	—	AT(10.04)	AAG(9.64)	AAAC(7.71)	AAATA(20.27)	AGAGGG(7.53)
	—	CG(0.21)	AGG(8.55)	AAGG(7.63)	AAAGG(5.52)	AGATAT(7.27)

Densities of SSRs and relative abundances of the different microsatellite length classes (i.e., mono-, di-, tri- up to hexa-nucleotides) across the different regions of the giant panda genome are presented in Table 3. SSRs were more abundant in intergenic regions (413,585 SSRs) than in introns (270,247 SSRs) and TEs (243,474 SSRs). More than 40% of different length categories of microsatellites were distributed in intergenic regions. For the microsatellites in CDSs, over 80% of them were tri- and hexa-nucleotides. Rare penta-nucleotides and hexa-nucleotides were found in 5′UTRs, CDSs or 3′UTRs.

**Table 3:** **Number, percentage, and relative abundance of SSRs in the different regions of the giant panda genome**

**Regions**		**5′UTR**	**CDS**	**Intron**	**3′UTR**	**Transposable elements**	**Intergenic regions**	**Total**
Genome size(Mb)		0.58	33.05	652.3	2.55	865.57	745.46	2299.51
Percentage of the genome		0.03	1.44	28.37	0.11	37.64	32.42	100.00
Mono	No.	39	110	145406	473	101984	200509	448521
	%^a^	0.01	0.02	32.42	0.11	22.74	44.70	100.00
	No./Mb	67	3	223	185	118	269	195
Di	No.	10	22	64574	103	80910	101809	247428
	%	0.00	0.01	26.10	0.04	32.70	41.15	100.00
	No./Mb	17	1	99	40	93	137	108
Tri	No.	47	969	9778	30	8048	19237	38109
	%	0.12	2.54	25.66	0.08	21.12	50.48	100.00
	No./Mb	81	29	15	12	9	26	17
Tatra	No.	5	15	43271	35	48745	76573	168644
	%	0.00	0.01	25.66	0.02	28.90	45.41	100.00
	No./Mb	9	0	66	14	56	103	73
Penta	No.	1	5	6589	10	3099	14287	23991
	%	0.00	0.02	27.46	0.04	12.92	59.55	100.00
	No./Mb	2	0	10	4	4	19	10
Hexa	No.	1	45	629	2	688	1170	2535
	%	0.04	1.78	24.81	0.08	27.14	46.15	100.00
	No./Mb	2	1	1	1	1	2	1
All SSRs	No.	103	1166	270247	653	243474	413585	929228
	%	0.01	0.13	29.08	0.07	26.20	44.51	100.00
	No./Mb	178	35	414	256	281	555	404

%^a^ = no./total no. of microsatellites in one kind of motifs.

**Figure 1:**
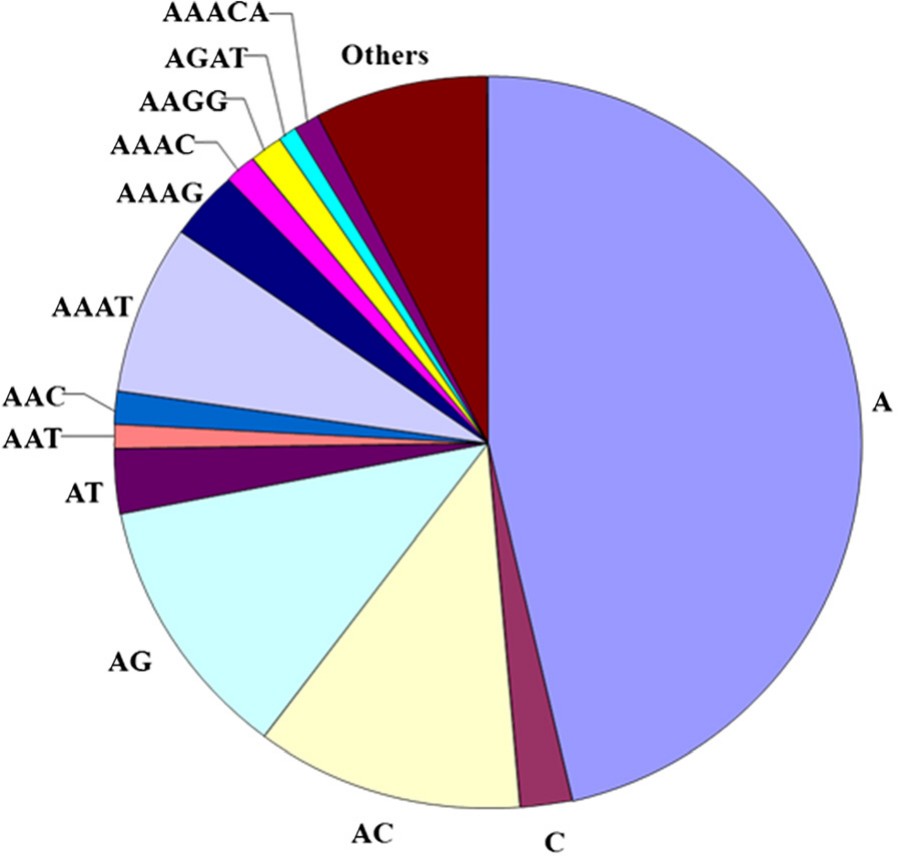
**Frequency of microsatellite motif categories in genome of giant panda (the 13 most frequent microsatellite motifs are shown in divisions).**

**Figure 2:**
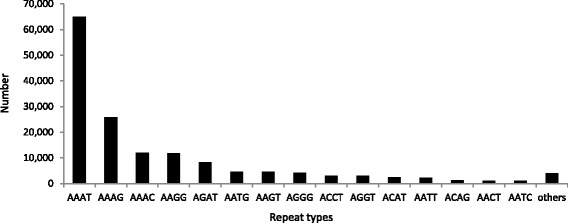
**The number distributions of each repeat copy categories in tetranucleotide.**

The 13 most abundant microsatellite classes were An, Cn, (AG)n, (AC)n, (AT)n, (AAC)n, (AAT)n, (AAAT)n, (AAAG)n, (AAAC)n, (AAGG)n, (AGAT)n, (AAACA)n, (Figure 1). Together, they comprised 90.1% of all microsatellites identified. For the tetra-nucleotides, the number distributions of each repeat motif were summarized in Figure 2 and comprised 97.42% of all of the tetra-nucleotides microsatellites identified in giant panda genome.

### Development of microsatellite markers

There were 154,677 tetranucleotide microsatellites sequences identified in the giant panda genome. Following the selection criteria, a total of 3,280 ‘potentially amplifiable loci’ with a repeat number in the range of 10 to 22 were isolated. A total of 336 candidate sequences, which were suitable for primer design (i.e., the flanking sequences should long enough and not be single-copy sequences), were chosen to develop an integrative database of tetranucleotide microsatellite markers for the giant panda. We designed and synthesized 160 pairs of primer for amplification which targeted as many of the SSR motifs as possible (see Additional file 1: Table S1). After amplification, the 61 loci that all showed a single band of expected size were further considered. All the forward primers of these 61 loci were labelled with different fluorescent dyes and then used to genotype 22 captive giant pandas (Chengdu, blood DNA). Loci that failed to provide clear signals in the expected size range or that lacked polymorphism were not considered further. Finally, 54 novel tetranucleotide microsatellites loci were discovered for the giant panda (see Additional file 2: Table S2).

The sequence raw data of these 54 loci were added to NCBI (GenBank accession numbers KF907130–KF907183). Additional file 2: Table S2 presented the characteristics of the 54 tetranucleotide microsatellites discovered in this study. A total of 246 alleles were identified and the number of alleles per locus ranged from 2 to 10. The observed and expected heterozygosities at each locus ranged from 0.091 to 0.909 and from 0.089 to 0.873, respectively (see Additional file 2: Table S2). The PIC ranged from 0.083 to 0.836 with an average of 0.533. We used the Micro-Checker software [28] to estimate the presence of genotyping errors such as null alleles, large allele dropouts or stuttering. There was no evidence for large allele dropouts or null alleles in the data set, and all the loci were neutral, which indicated that the data were sufficient for further analysis. The failure of loci to meet HWE will have an effect on population genetic analysis. HWE test in this study indicated that 3 out of the newly discovered 54 loci deviated significantly from HWE (P < 0.01, see Additional file 2: Table S2) and, therefore, should be discarded. LD also influences population genetic analysis. However, it is not clear whether the 54 new tetranucleotide microsatellites loci are on different chromosomes due to the fact that the genome sequences of the giant panda were assembled into scaffolds but not annotated to different chromosomes. Loci located in different scaffolds were the first choice in order to reduce the influence of LD to a low level (see Additional file 3: Table S3).

### A universal genetic marker system based on microsatellites

The remaining 51 novel polymorphic tetranucleotide microsatellite loci were further tested to establish the universal genetic marker systems for the giant panda. Considering that the system will be used for the wild giant panda, these standard loci must be applied to non-invasive samples. Therefore, 30 faecal DNA samples from captive giant pandas (Chengdu) were used to test the sensitivity and quality of the 51 loci. However, the amplification success rates of 9 loci (GPL-10, GPL-21, GPL-26, GPL-37, GPL-58, gpz-11, gpz-25, gpz-3, gpz-40) were less than 50% faecal, which means that these loci showed a lack of responsiveness to faeces and, therefore, were not suitable for faecal DNA analysis. Another 10 loci (GPL-1, GPL-7, GPL11, gpz-50, GPL-75, gpz-36, gpz-26, gpz-48, gpz-50, gpz-55) were rejected due to a lack of polymorphism. What’s more, repeat tests indicated that multiple amplification or false amplification existed in 16 loci. The genotypes of the remaining 16 loci were compared between faecal DNA and blood DNA (n = 15) for a further stability analysis. Fortunately, all loci except one (GPL-12) were confirmed stable and reliable because no difference was found in genotypes of the 15 pairs of matched samples, which indicated there is no genotyping error for the remaining 15 loci. The test about the relationship between exposure time of faecal samples and the stability of the loci showed that these loci can be used to the faecal samples with even five weeks exposure to the wild environment. Especially, the loci gpz-47 and gpz-06, which can be most easily amplified in the PCR, were the most stability and responsive loci in the 15 markers. The 15 markers (Table 4), which showed high levels of polymorphism, strong stability, and good repeatability, were used to genotype the rest of the 27 faecal samples and to build a data base for Chengdu captive giant pandas (n = 57).

**Table 4:** **Characteristics of the novel microsatellite marker system and the genetic diversity of Chengdu captive giant panda population, including locus names, primer sequences, accession number, repeat unit, fluorescent dyes, annealing temperatures (Tm), length (bp), numbers of individuals genotyped (N), numbers of alleles(k), observed heterzygosity (HObs), expected heterzygosity (HExp), allelic richness (A**_R_ **), Polymorphism Information Contents (PIC), HWE** ***P*** **values (P-value)**

**Locus name**	**Primer sequences(5′—3′)**	**Accession no.**	**Repeat unit**	**Fluorescent dyes**	**Tm (°C)**	**Length (bp)**	**N**	**k**	**HObs**	**HExp**	**A**_**R**_	**PIC**	**P-value**
gpz-6	F: CCTGGCAGGGCAAAGTATT	KF907161	(AAAG)11	FAM	60	202	56	6	0.714	0.689	6.000	0.633	0.2615
	R: CCCCGTGAAAACATCAAGAC												
gpz-47	F: GACCTCAGTGTACGCCCAGT	KF907176	(AATG)20	TAMRA	60	230	57	4	0.544	0.524	4.000	0.468	0.3557
	R: CTGGACAGGCAGGTAGAAGC												
gpz-20	F: CCCTCTCGTTGTGTCTCTCTG	KF907169	(AAAG)10	FAM	63	248	52	10	0.731	0.724	10.000	0.695	0.1302
	R: CACCTGGTAAATGGCACCTT												
GPL-47	F: TCCCCCTCTATGGTAAAAGG	KF907147	(TCTA)20	FAM	65	180	53	6	0.849	0.819	6.000	0.783	0.2161
	R: CCATGTTGGGTGTAGGGATT												
GPL-29	F: TCCAAGGCTTCAAACAAGGT	KF907139	(ATCC)19	TAMRA	60	215	56	4	0.714	0.677	4.000	0.617	0.2800
	R: CACCACAGGTGCCAATTATG												
GPL-60	F: TGCCGGAAAGTTCTAAGCAT	KF907152	(TCTT)12	FAM	63	218	57	5	0.702	0.719	5.000	0.668	0.3991
	R: TTTCTCTCCCTCTCCCCTTC												
gpz-54	F: CAATATTTTAAGGCGTGGGACT	KF907181	(AGAT)18	TAMRA	63	245	56	5	0.714	0.704	4.929	0.643	0.6226
	R: GCATAATTGCAGAACCAGAGC												
GPL-8	F: TGGTTTTGCAAGGATGACAG R:TTGTGACAAGCAAGCTCCAC	KF907132	(ATCC)11	HEX	63	248	54	4	0.648	0.639	4.000	0.584	0.4311
GPL-31	F: GCATCCTTGTCCTCTTGGAG	KF907141	(ATCT)21	FAM	60	183	57	3	0.632	0.585	3.000	0.490	0.1640
	R:GCATTGTTTTCTACTCTACAAATATCC												
GPL-44	F: TTCTCCCTCTGTCTGCCACT	KF907146	(ATAA)21	FAM	63	232	53	3	0.491	0.525	3.000	0.461	0.2543
	R: ACCATTCTGGGTGCGATAAC												
gpz-51	F: GGGGAGGATATGTGTTGTGG	KF907179	(AGAT)11	TAMRA	60	175	57	4	0.579	0.503	3.993	0.424	0.0440
	R: TGCTTTGGATTTATTGGAGCA												
GPL-28	F: GAAAGAAGGGCAGGGATAGG	KF907138	(ATAA)21	FAM	63	238	56	3	0.536	0.486	2.995	0.382	0.2594
	R: TGACCAAGAACTCACGGTTG												
GPL-53	F: CCAGAAAATGGCTTTCATGC	KF907148	(ATTT)21	HEX	65	210	55	6	0.382	0.380	5.997	0.362	0.6395
	R: TCTCTTTCTCTGCCCCACAC												
gpy-20	F: GCAGGCACTCAAGAGGTGTT	KF907159	(TTTG)16	TAMRA	63	197	56	3	0.482	0.492	3.000	0.439	0.8984
	R: CCTTGTGCTAAACACAGGTGA												
gpy-5	F: CTCGGGAGCTTTGTACCATC	KF907157	(AACT)16	HEX	63	228	57	4	0.509	0.510	3.993	0.459	0.4341
	R: CAGAGAGCCCAAACCTCAAC												
Mean								4.7	0.615	0.598	4.660	0.541	---

### The establishment of a universal individual identification method

**Figure 3:**
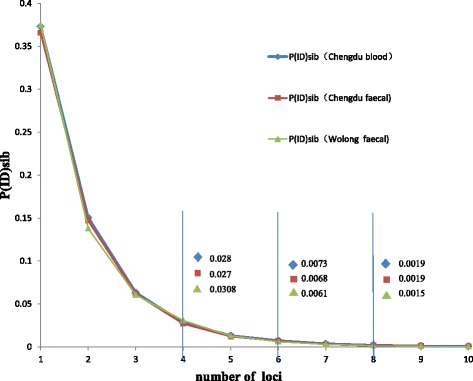
**A plot showing the effect of number of microsatellites on the probability of identity assuming all individuals are siblings PID(sibs) for a set of Chengdu and Wolong captive samples (Chengdu blood, n = 22; Chengdu faecal, n = 57; Wolong faecal, n = 61).**

As one of the applications of this microsatellite system, a universal individual identification method was established by the present study. We know that loci which are higher in expected heterozygosity (He) are more useful for individual identification. The 15 loci selected range in He a high of 0.819 to 0.380 (Table 4) (the first two were the most stability and responsive loci in this study). The number of microsatellite markers used for individual identification is extremely important because it has consequences for all subsequent analyses [17,29]. Too many markers can increase genotyping errors, false genotypes, and overestimations of population sizes [30]; while too few or insufficient markers would lead to underestimations [31]. Therefore, for the purpose of individual identification, the question is how many of the loci should be used? The earlier measure of probability of identity (PID) developed by Waits et al. [32] was preferred for individual identification; however, PIDsib (estimating PID among sibs) is a more conservative minimum number of loci necessary to distinguish individuals with PIDsib value <0.01 [33]. In order to determine the minimum number of loci required for accurate individual identification of giant pandas, besides the most stability and responsive two loci (gpz-47 and gpz-06), we first investigated how the PIDsib values for the giant panda samples change as the number of loci are increased according to the He of other 13 loci. Using GIMLET program [34], we calculated PID(sibs) curves based on 10 loci for the captive populations using different kinds of samples (Chengdu blood = 22, Chengdu faecal = 57). The PID(sibs) curves of the first two loci were gpz-47 and gpz-06, then we added the loci set with the most informative loci one by one. It revealed that the subset of six loci was enough for accurate individual identification (PIDsib < 0.01) (the first six loci in Table 4) (Figure 3).

Individual identification simulations were conducted with the six loci using the CERVUS 3.0 software [35]. The result indicated that the set of six loci is effective for individual identification in the 57 captive giant panda. Furthermore, an individual identification test was first conducted using 13 captive panda faecal samples without known information. The genotype result indicated that the 13 faeces came from 11 giant pandas, which was in agreement with the record (see Additional file 4: Table S4). What’s more, these 60 faecal samples from wild giant pandas in the Wolong Nature Conservation Centre were identified as 22 unique individuals, which revealed that the method is also effective in wild faecal samples (see Additional file 5: Table S5).

### Genetic diversity of Chengdu captive population

The genetic diversity of Chengdu captive giant panda population was analyzed using 15 tetranucleotide microsatellite loci. As showed in the Table 4, a total of 70 alleles were identified among the 57 giant pandas. The number of alleles per locus ranged from three (GPL-28, GPL-44, GPL-31, gpy-20) to a maximum of ten (gpz-20). Allelic richness (*A*R) at each locus ranged from 2.995 to 10.000 alleles. The mean *A*R (mean *A*R = 4.660) in this study is much higher than that of Shen et al. (mean *A*R = 3.957). The observed and expected heterozygosity ranged from 0.382 to 0.849 and from 0.380 to 0.819, respectively. Mean Ho and He were 0.615 and 0.598, respectively, which were both a little lower than that of Shen et al. (2009) (Ho = 0.671, He = 0.634). A large variation in heterozygosity was observed in different loci. The mean polymorphic information content (PIC) was 0.541 (ranging from 0.362 to 0.783) in this captive population. HWE tests revealed that none of the loci deviated from HWE in this captive populations (P > 0.01).

## Discussion

### Genome-wide distribution and organization of microsatellites in the giant panda

In this study, we characterized the SSRs in the entire genome sequencing assembly of giant panda and analyzed their frequency and distribution in different genomic regions. Most of the SSRs are mono-, di- and tri-nucleotides, accounting for up to 75% of all of the SSRs identified. The distribution of microsatellites in the giant panda was agreement with Li et al. [36], which reported that di-nucleotides are the most common microsatellites in many organisms without taking into account of mononucleotide repeats. In most genomes, motifs with short repeated units (mono- to tri-nucleotides) were more abundant than long repeated units, indicating that longer repeats correlate with higher instability [37].

Moreover, SSRs identified in the different regions provided useful information about possible physical linkage between microsatellite loci. The highest SSR relative abundance was found in intergenic regions, followed by introns. The findings in this study are in agreement with the prior studies that the majority of SSRs are embedded in non-coding DNA, either in the intergenic sequences or introns [38]. Although the relative abundance of SSR in exons was lowest, there was a propensity of tri- and hexa-nucleotides in exons, which was consistent with Labbe et al. [39] and Qian et al. [37]. Such a propensity may be to suppress the other categories of SSRs, thus reducing the incidence of frameshift mutations in coding regions caused by nontriplet repeats [39,40].

The genome-wide distribution and organization of SSR highlighted a non-random distribution of these repeats which may be involved in the genome plasticity. The wealth motifs of genome-wide SSR markers identified in the present study now opens new perspectives for the development of a wide range of microsatellite markers in the panda genome. Especially, these tetranucleotide microsatellite data obtained in this study will be helpful in developing SSR markers that could be applied in the establishment of a universal marker system.

### The establishment of a novel microsatellite marker system

In recent years, more and more researchers have become aware of the problem of microsatellite data quality and its consequence for population analyses [20,41,42]. Highly polymorphic microsatellite markers could easily suffer from mutations, allelic dropouts, undetectable null alleles [43] and genotyping errors [20,43,44]. If null alleles exist in an SSR marker, an intrinsically heterozygotic individual might be misinterpreted as homozygotic, leading to inaccurate and biased genetic estimates [45]. Except null alleles, researchers should be aware of the selective neutrality, Hardy–Weinberg equilibrium (HWE) and Linkage disequilibrium (LD) [46]. The different parts of the genome have differences in the mutation rate and the accepted selection pressure. The microsatellites may vary in these aspects. The 15 loci we selected in this study all showed neutrality, no null alleles and no deviation from Hardy–Weinberg equilibrium (HWE), which ensured these loci were available and effective.

The instability microsatellite markers, which is easily produce error genotypes, may result in mistakes in the individual identification, paternity test, population structure and genetic diversity analysis for the different species [47]. Especially for the DNA samples with poor quality, it may produce more error genotypes when using these instability loci. In this study, we used the *in silico* approach to screening the whole genome sequence of the giant panda and selected the most stability loci from the large number of tetranucleotide microsatellite sequences. We designed 160 tetranucleotide microsatellite primer pairs, in which 51 novel loci showed good polymorphic, selectively neutral, no deviation from Hardy–Weinberg equilibrium (HWE) and high stability were selected based on blood DNA samples. However, one of the great challenges in the research of giant panda is that it is extremely difficult to get the good samples. The blood collection process of captive giant pandas is very complicated and may have adverse effects on their health, which raises questions of research ethics for both the scientific community and general public. It is even harder to obtain blood and muscle samples from wild giant pandas. Non-invasive genetic sampling, where DNA is recovered from discarded sources such as shed hair and faeces [48], is a necessary alternative to tissue sampling of giant panda. While high concentrations and high quality of DNA from non-invasive samples will greatly reduce genotyping errors such as allele dropouts or false alleles in genetic studies [17-20], they are very difficult to obtain. In all studies in which typing errors were checked, a non-negligible error rate from 0.2% to more than 15% per locus was reported [20]. Even higher error rates are known to occur in studies using DNA with poor quality or low concentrations [17,18], as is in the case of non-invasive genotyping. Therefore, the loci used to establish the novel microsatellite marker system must show a lower error rate and be responsive to non-invasive DNA. In the previous studies, most of the loci were screened with blood or muscle DNA but nearly never tested with non-invasive samples for responsiveness, which resulted in a large number of wild faecal samples being abandoned due to failed amplification in PCR. In this study, the screening procedures for these novel 15 high stability and repeatability loci were relatively rigorous. First, non-invasive samples were used to test the sensitivity of the inferred markers, which ensured that the loci were responsive to DNA with low quality and concentrations. In addition, the repeat tests conducted by faecal DNA guaranteed the stability and reliability of the selected loci, which reduced the probability of genotyping error at the loci level. Moreover, the relationship test between the exposure time of faecal samples and the stability of the loci indicated that these loci could be used in the wild samples with an exposure time of five weeks. Therefore, these 15 loci with high stability and repeatability will be widely and effectively used in future studies.

### The application of the novel microsatellite marker system

Based on the 15 loci, we established the genotype database of the Chengdu captive giant pandas. The database displayed the size range of alleles characteristic of different loci, which facilitates the accurate identification of genotypes in future studies. This database contains the basic genetic information of microsatellites for Chengdu captive giant pandas, which can be shared with other researchers to allow broader application. Moreover, we would like to accept more genetic information of other populations of giant pandas and make much more improvements for this data. Also, it is much convenient to compare the genetic diversity of different populations and to understand the population structure using the universal genetic markers for the giant panda.

Although China has taken three national surveys to estimate the population size of wild giant panda and the millions of dollars already spent on, the number is still controversy in the researchers. Microsatellite analysis using faecal DNA has proven effective in estimating population size of elusive animals while the error genotypes in different loci may result in large deviation from the real result. Too many markers can increase genotyping errors and overestimations of population sizes [30] while too few or insufficient markers would leading to underestimations [31]. Previous studies indicated that a single-locus error rate of 1% would add up to 10% using ten loci [17]. Considering the maximum threshold of 5% of genotyping errors in population size estimation [49], it could be one means to minimize potential error sources by reducing the number of microsatellite markers used. In most other studies on wildlife forensics, six to ten microsatellite markers are commonly used [50-52]. In any case, the sufficient discriminating power must be contained in the minimum subset of microsatellite loci needed for accurate individual identification [31,47]. Following Waits et al. [33], the value of PIDsib was used as a bound to estimate the minimum number of loci necessary to distinguish between individuals. A subset of six microsatellite loci in our study was enough for accurate individual identification in giant pandas (PIDsib < 0.01) in this study. The individual identification test for faeces from captive and wild pandas were further indicated that this subset of loci is available and quite effective in making accurate individual identifications. Moreover, we would like to encourage using this method to establish a shared wild giant panda microsatellite database to facilitate and enhance further research on the giant panda. All researchers could add the data of new individuals to the database. Genetic information about this species would accumulate more rapidly, which would be more convenient for researchers based in different sites to study important ecology problems for wild giant pandas (such as population size, population dynamics, breeding behaviour, habitat use, and home range size).

Besides individual identification, genetic diversity of Chengdu captive giant panda population was also analysed as another application of the marker system. It demonstrated that these markers developed in this study were effective in genetic diversity analyses. Moreover, the mean allelic richness of the Chengdu captive population in our study was much higher than Li et al. [8] and Shen et al. [7]. However, the level of heterozygosity was similar, which means that the loci developed in this study with a higher number of alleles. While, one of the aim of the conservation programs is that to conserve genetic diversity over long periods as genetic diversity is essential to ensure the conservation of the evolutionary potential which allows the population to adapt to changing environments [53]. Therefore, monitor the genetic diversity using high quality markers in different populations are needed in order to the long-term persistence of this species.

## Conclusions

This analysis of microsatellites in completely sequenced panda genome provides a snapshot of the differential coverage and density of 1–6 bp repeats in this species. In particular, the mono-, di- and tri-nucleotides repeats are accounting for up to nearly 75% of all of the SSRs identified. The majority of SSRs were embedded in non-coding DNA and there was a propensity of tri- and hexa-nucleotides in exons. Especially, we focused on the 154,677 tetranucleotide microsatellites because they were much more accurate and reliable than di- and tri-nucleotide microsatellites. The final 51 novel polymorphic tetranucleotide microsatellite loci were further used to establish the universal genetic marker system for giant pandas with faecal samples. The individual identification method, which is established based on these loci, is particularly effective in assessing the population size for wild giant pandas. Moreover, the effectively of this marker system in analyses the genetic diversity of one captive giant panda population will promote other population studies. Undoubtedly, the development of large sets of markers should in turn facilitate population genetic research on giant panda.

## Methods

### Sample collection and DNA preparation

Faeces and blood samples were collected from the Chengdu Research Base for Giant Panda Breeding (Chengdu, faeces = 57, blood = 22) and the China Research and Conservation Centre for the Giant Panda in the Wolong Nature Reserve, Sichuan Province (Wolong, faeces = 61). These animals included close relatives such as siblings, which were necessary for standardization of the final set of loci used for individual identification. Matched samples from blood and fresh faeces (n = 15) were included in the samples from Chengdu for the stability analysis of the markers. Captive faecal samples (*n* =13) without any prior background information were collected from Chengdu for individual identification tests. Wild giant panda faecal samples (*n* = 60) were collected from Wolong Nature Reserve at the beginning of 2013. In order to reduce the chance of sampling from the same individual, different samples were not collected from the same home range [6]. In addition, all samples were GPS recorded and mapped using Arcview 3.2a.

All blood samples were obtained from yearly routine blood tests for panda health. All samples were collected in accordance with the regulations for the implementation of China on the protection of terrestrial wild animals (State Council Decree [1992] No.13) and were approved by Wildlife Protection Office, Sichuan Provincial Forestry Departments (China). Blood and faecal samples were carefully collected to avoid contamination and preserved in EDTA Vacutainers and sterile bags, respectively. All samples were frozen at −20°C. Total genomic DNA extracted from blood and faecal samples were performed using the commercially available Qiagen DNeasy Blood & Tissue Kit and Qiagen QIAamp Stool Mini Kit respectively, according to the manufacturer’s instructions with some optimizations [54].

### Genome sequences and SSR identification

The entire genome of the giant panda was directly downloaded from UCSC Genome Bioinformatics (http://genome.ucsc.edu/). The sequences of the gene models, introns, coding sequences (CDSs), 5′ untranslated regions (5′ UTRs), 3′ untranslated regions (3′ UTRs), transposable elements (TEs) and intergenic regions were generated according to the positions in the genome annotations. The intergenic regions referred to the genomic regions that were not included the introns, CDSs, UTRs or TEs. Genome sequences were scanned for microsatellite content using the program MSEA v2.3 (http://code.google.com/p/msdb) [55]. Detection criteria were restricted to identify perfect SSRs (i.e., those with uninterrupted repeats and compound motifs) of 1–6 bp and a minimum repeat number of 12, 7, 5, 4, 4, and 4, for mono-, di-, tri-, tetra-, penta- and hexa-nucleotide microsatellites, respectively [56]. Repeats with unit patterns being circular permutations and/or reverse complements were considered as one type in this study [27,57]. For example, the AGG contains AGG, GGA, GAG, CCT, CTC and TCC in different reading frames or on the complementary strand. To facilitate the comparison among different repeat categories or genomic regions, the relative abundance, which means the SSR number per Mb of the sequence analyzed, and the relative density, which means the SSR length (in bp) per Mb of the sequence analyzed, were introduced [37,58].

### Development of SSR markers

The flanking regions of microsatellites (200 bp either side) were extracted from the program output in order to design the primer sets for the microsatellite loci identified. These output sequences were further manually scanned and filtered according to the criteria of microsatellite identification which are as follows: (1) repeats should be tetranucleotide repeats; (2) microsatellites should not be in published repeat sequences; (3) the number of repeats should be in the range of 10–22; (4) the flanking sequences of microsatellites should not be single-copy sequences but must be long enough to design primers (i.e., more than 20 bp). A large number of 400-500 bp sequences containing a tetranucleotide microsatellite of interest were extracted and compared with the previous published 15 tetranucleotide microsatellite sequences using the software Clustal_X 1.83 [59]. We used Primer 3 [60] to design the primers to amplify the selected sequences. The lengths of the primers designed in the present study were between 17 and 27 bp, with a maximum of three degenerated positions and with an expected product size between 100 and 400 bp. We then tested the primers for reproducible amplification in three giant pandas under the standard PCR conditions, with annealing temperatures altered according to the primer sequence. During optimization, we tested whether amplification was improved by the addition or decrease of MgCl_2_, or by a higher or lower annealing temperature.

### Polymorphism microsatellite isolation

After optimization, the primers with single band of expected size in the amplification were selected to label with one of three fluorescent dyes (FAM, TAMRA or HEX) (Invitrogen Shanghai Sangon Biological Engineer Technology & Services, Shanghai, China) in the forward primers for fragment analysis on Applied Biosystems 3100 Genetic Analyzers. The blood DNA from 22 captive giant pandas was used to evaluate the ability of the primer pairs to amplify polymorphic bands. PCR amplifications were carried out in 25 μL reaction mixtures, comprising approximately 50 ng of template DNA, 1.5-2 mm MgCl_2_ (TaKaRa, Japan), 200 μm of each dNTP, 15pmol of each primer, and 0.3 U of Ampli *Taq* DNA polymerase (TaKaRa, Japan). Amplifications were performed using the following PCR procedure: an initial denaturation step for 5 min at 95°C, followed by 35 cycles of 95°C for 45 s, 30 s at locus-specific annealing temperature (55°C--65°C) and 50 s at 72°C, and a final elongation for 10 min at 72°C. For genotyping, the PCR amplification products were separated by capillary electrophoresis using a denaturing acrylamide gel matrix on an ABI PRISM 377 Genetic Analyser (Applied Biosystems) using GeneScan Tarmara 350 internal size standard (ABI). Alleles were detected using the GeneScan⁄Genotyper software package of Applied Biosystems. Markers which have a strong tendency to form stutter peaks were excluded in this step. The remaining markers were taken into consider with amplification if (a) the expected PCR products were observed for more than 90% of the 22 samples investigated and (b) the number of bands did not exceed the ploidy of any individuals sampled (diploid in the giant panda). What’s more, microsatellites fragments were considered as in expected size if their length was within ±30% of the target sequence length.

### High sensibility polymorphism microsatellite

The faecal DNA from 30 captive giant panda was used to test whether the polymorphic markers showed highly responsive and could be applied to faecal DNA. In order to control the inhibiter in the faecal DNA, the bovine serum albumin (BSA) was added in the PCR mixture. The markers would be excluded if (a) the amplification success rate less is than 50% in faecal samples and (b) having many stutter peaks as in the blood samples above. Meanwhile, the ‘multi-tube procedure’ [18] was used to test the tendency for genotyping errors in these microsatellite loci.

### The stability of these microsatellites

The genotyping results of blood and faecal DNA obtained from the same panda (15 pandas in total) were compared to evaluate the reliability and the stability of these microsatellites. Moreover, in order to test whether these loci can be used to the faecal samples with a long exposure to the wild environment (time gradient: one to seven weeks), the relationship between the exposure time and the stability of the loci were tested in the present study.

### The application of the novel microsatellite marker system

The markers which showed good polymorphism, repeatability and stability in both blood DNA and faecal DNA were used to genotype the rest of the 27 faecal samples and to build a database for the Chengdu captive giant pandas (n = 57). Furthermore, we established a universal individual identification method based on the novel set of genetic markers. We also analysed the genetic diversity Chengdu captive giant panda population.

### Statistical and genetic data analysis

We used Micro-Checker software [28] to estimate the presence of genotyping errors such as null alleles, large allele dropout, or stuttering in the data set. The number of alleles (A), observed heterozygosity (Ho), expected heterozygosity (He), polymorphic information content (PIC), and the paternity test were calculated with the software of CERVUS 3.0 [35]. Deviations from Hardy–Weinberg equilibrium (HWE) and linkage disequilibrium (LD) were tested for by using GENEPOP 3.4 [61]. To test the discrimination power of sets with different numbers of microsatellites, the probability of pairs of individuals bearing an identical multi-locus genotype (P(ID)) was computed using GIMLET 1.3.1 [34]. Since PIDsib is a more conservative P(ID) for full sibs, we used PIDsib as an upper limit to the probability that pairs of individuals would share the same genotype. Individual identification was analysed by CERVUS 3.0 [35].

### Data accessibility

DNA sequences: GenBank accessions KF907130–KF907183; see Additional file 2: Table S2 for details.

## Additional files

Additional file 1: Table S1. The information of the 160 primer pairs. Additional file 2: Table S2. Characteristics of 54 polymorphism microsatellites developed in this study. Shown are locus names, primer sequences, accession number, repeat units, fluorescent dyes, annealing temperatures (Ta), length (bp), numbers of individuals genotyped (*N*), numbers of alleles(A), observed heterzygosity (*H*O), expected heterzygosity (*H*E), Polymorphism Information Contents (PIC), HWE *P* values (P-value). Additional file 3: Table S3. The linkage disequilibrium (LD) analysis between the 54 microsatellite loci. Additional file 4: Table S4. The genotype result of the 13 captive giant panda faeces based on 6 microsatellites. Additional file 5: Table S5. The genotype result of the 22 unique wild giant panda individuals based on 6 microsatellites.
