# Supplementary material for: Genome-wide survey and analysis of microsatellites in giant panda (Ailuropoda melanoleuca), with a focus on the applications of a novel microsatellite marker system
Source: BMC Genomics. 2015 Feb 7;16(1):61. doi: 10.1186/s12864-015-1268-z (PMC4335702; doi:10.1186/s12864-015-1268-z)
Supplement: Additional file 2: Table S2. — Characteristics of 54 polymorphism microsatellites developed in this study. Shown are locus names, primer sequences, accession number, repeat units, fluorescent dyes, annealing temperatures (Ta), length (bp), numbers of individuals genotyped (N), numbers of alleles(A), observed heterzygosity (HO), expected heterzygosity (HE), Polymorphism Information Contents (PIC), HWE P values (P-value). [file 12864_2015_1268_MOESM2_ESM.doc]

Table S2 Characteristics of 54 polymorphism microsatellites developed in this study. Shown are locus names, primer sequences, accession number, repeat units, fluorescent dyes, annealing temperatures (Ta), length (bp), numbers of individuals genotyped (*N*), numbers of alleles(A), observed heterzygosity (*H*O), expected heterzygosity (*H*E), Polymorphism Information Contents (PIC),HWE *P* values (P-value)

| Locus name | Primer Sequences（5’—3’） | Accession No. | Repeat unit | Fluorescent dyes | Tm(℃) | Length(bp) | N | A | HObs | HExp | PIC | P-value |
| --- | --- | --- | --- | --- | --- | --- | --- | --- | --- | --- | --- | --- |
| GPL-1 | F:TATCAGCAACACCCATGAGC  R:CCAAAATCAAGGTGTCAGCA | KF907130 | (AAAG)13 | FAM | 63 | 169 | 22 | 2 | 0.227 | 0.426 | 0.330 | 0.0397 |
| GPL-7 | F:ACACACGGAATGACAAAGCA  R: CCCATTTTCCATTACCCAGA | KF907131 | (ATAA)20 | TET | 63 | 218 | 22 | 3 | 0.545 | 0.549 | 0.468 | 0.5465 |
| GPL-8 | F: TGGTTTTGCAAGGATGACAG  R:TTGTGACAAGCAAGCTCCAC | KF907132 | (ATCC)11 | HEX | 63 | 248 | 22 | 4 | 0.591 | 0.625 | 0.553 | 0.4947 |
| GPL-10 | F: CCACACTGCAATGACCTGAT  R:GTGCCACAACTGATTGAAGC | KF907133 | (ATCT)11 | FAM | 63 | 239 | 18 | 6 | 0.278 | 0.787 | 0.732 | 0.0000 |
| GPL-11 | F: CTGGCATAGAGTGGGTGCTT  R:AGGTCAGTGGCTCAAGAGGA | KF907134 | (ATGA)20 | TET | 63 | 231 | 21 | 3 | 0.333 | 0.361 | 0.315 | 0.4693 |
| GPL-12 | F: CCCCCATTGTTAACATCCTG  R: CCCTGCTCGCACTCATAAAT | KF907135 | (ATTC)20 | FAM | 55 | 188 | 22 | 4 | 0.727 | 0.671 | 0.589 | 0.2646 |
| GPL-21 | F: TCACACTACCCTTGCTGGTG  R:TGTGCCATGAACTCATTACTCTG | KF907136 | (AAAG)22 | FAM | 60 | 208 | 22 | 9 | 0.909 | 0.873 | 0.836 | 0.1849 |
| GPL-26 | F: AGGGCCCAGCACAATAAATA  R: TGGAAGCAACAGTGTCCATT | KF907137 | (AGAT)20 | HEX | 60 | 250 | 22 | 4 | 0.636 | 0.618 | 0.555 | 0.5345 |
| 1GPL-28 | F: GAAAGAAGGGCAGGGATAGG  R: TGACCAAGAACTCACGGTTG | KF907138 | (ATAA)21 | FAM | 63 | 238 | 21 | 2 | 0.571 | 0.455 | 0.346 | 0.2371 |
| GPL-29 | F: TCCAAGGCTTCAAACAAGGT  R: CACCACAGGTGCCAATTATG | KF907139 | (ATCC)19 | TAMRA | 60 | 215 | 22 | 4 | 0.773 | 0.667 | 0.598 | 0.1484 |
| GPL-30 | F: ATGGTGAACGGCTAAGTTGG  R: TGATGTATGGTATGGTGACTAACA | KF907140 | (ATCT)21 | HEX | 63 | 205 | 21 | 4 | 0.714 | 0.749 | 0.681 | 0.4710 |
| GPL-31 | F: GCATCCTTGTCCTCTTGGAG  R:GCATTGTTTTCTACTCTACAAATATCC | KF907141 | (ATCT)21 | FAM | 60 | 183 | 21 | 3 | 0.571 | 0.591 | 0.488 | 0.5472 |
| GPL-35 | F: TAGGGAGGTGGGAGCTTTTT  R: TGAGCCTCACTCTGACTCCA | KF907142 | (ATTT)21 | TAMRA | 63 | 184 | 22 | 7 | 0.864 | 0.725 | 0.666 | 0.0871 |
| GPL-37 | F: TCTGGCTTCTCTGGCTCAGT  R: TGAAAACAACACCAACGTTCA | KF907143 | (ATTT)20 | HEX | 60 | 218 | 22 | 4 | 0.727 | 0.627 | 0.533 | 0.1844 |
| GPL-38 | F: GAGATCGTTCACCTTCTTGGA  R: TGAATTCAGTAAAATGGCAAAA | KF907144 | (TCCT)24 | TAMRA | 60 | 246 | 22 | 4 | 0.091 | 0.551 | 0.491 | 0.0000 |
| GPL-40 | F: TGAGGGATAGCTTTGTTGCAT  R: GGCTGTCAGCTGATTCTTCA | KF907145 | (TCTT)10 | FAM | 60 | 170 | 22 | 7 | 0.773 | 0.805 | 0.756 | 0.4079 |
| GPL-44 | F: TTCTCCCTCTGTCTGCCACT  R: ACCATTCTGGGTGCGATAAC | KF907146 | (ATAA)21 | FAM | 63 | 232 | 22 | 3 | 0.409 | 0.413 | 0.366 | 0.1824 |
| GPL-47 | F: TCCCCCTCTATGGTAAAAGG  R: CCATGTTGGGTGTAGGGATT | KF907147 | (TCTA)20 | FAM | 65 | 180 | 20 | 6 | 0.800 | 0.812 | 0.761 | 0.4159 |
| GPL-53 | F: CCAGAAAATGGCTTTCATGC  R: TCTCTTTCTCTGCCCCACAC | KF907148 | (ATTT)21 | HEX | 65 | 210 | 21 | 5 | 0.524 | 0.505 | 0.464 | 0.6489 |
| GPL-55 | F: AATCAATCAGCTGGGTCCTG  R: CCTCGGGCTTCTACTCAGTG | KF907149 | (ATTT)20 | TAMRA | 65 | 246 | 20 | 4 | 0.550 | 0.629 | 0.560 | 0.1595 |
| GPL-57 | F: CCAAGGACAGCAGCAAAAAT  R: CACACACATTTTCCCTTCCA | KF907150 | (ATTT)15 | HEX | 63 | 200 | 18 | 3 | 0.444 | 0.414 | 0.363 | 0.5007 |
| GPL-58 | F: TTAGGGTGATGCTGGTTTCA  R: CCAGATGGTTTGTTTCACCA | KF907151 | (TCCT)12 | TAMRA | 63 | 245 | 20 | 7 | 0.900 | 0.833 | 0.787 | 0.1536 |
| GPL-60 | F: TGCCGGAAAGTTCTAAGCAT  R: TTTCTCTCCCTCTCCCCTTC | KF907152 | (TCTT)12 | FAM | 63 | 218 | 22 | 5 | 0.682 | 0.739 | 0.678 | 0.2837 |
| GPL-61 | F: TCACCCAACACTTACCAGGA  R: TGAAACCATTTTTACCTACCCAAT | KF907153 | (ATTT)22 | HEX | 60 | 246 | 22 | 6 | 0.818 | 0.726 | 0.666 | 0.4834 |
| GPL-75 | F: CCACTGACTGTTCGTTGGAA  R: TGGGCTCACACTCTCTTTCA | KF907154 | (ATTT)20 | TAMRA | 60 | 223 | 22 | 4 | 0.273 | 0.358 | 0.329 | 0.1190 |
| GPL-80 | F: TTCAGCTCTTTGCCATTCTTT  R: TGTCTTCCTCTGCCATTTCC | KF907155 | (ATTT)20 | TAMRA | 63 | 190 | 22 | 7 | 0.864 | 0.774 | 0.726 | 0.1032 |
| gpy-2 | F: TACCATGAACTGCCCATCAA  R: TGGGACTGGGAATTGAAAAA | KF907156 | (AAAC)20 | HEX | 63 | 246 | 21 | 5 | 0.667 | 0.684 | 0.614 | 0.4529 |
| gpy-5 | F: CTCGGGAGCTTTGTACCATC  R: CAGAGAGCCCAAACCTCAAC | KF907157 | (AACT)16 | HEX | 63 | 228 | 22 | 3 | 0.591 | 0.540 | 0.471 | 0.3479 |
| gpy-13 | F: CTTTGGTGGCCAAGAGAAAG  R: AAGCGTCCATGTTCCAGAGT | KF907158 | (TTAG)10 | TAMRA | 63 | 196 | 22 | 5 | 0.591 | 0.678 | 0.599 | 0.1750 |
| gpy-20 | F: GCAGGCACTCAAGAGGTGTT  R: CCTTGTGCTAAACACAGGTGA | KF907159 | (TTTG)16 | TAMRA | 63 | 197 | 22 | 3 | 0.545 | 0.595 | 0.516 | 0.2013 |
| gpz-3 | F: TCATTGCATGGCCTTATTTG  R: TGCGTTTGCGGTTAATGATA | KF907160 | (AAAG)13 | FAM | 63 | 168 | 21 | 6 | 0.524 | 0.722 | 0.654 | 0.0273 |
| gpz-6 | F: CCTGGCAGGGCAAAGTATT  R: CCCCGTGAAAACATCAAGAC | KF907161 | (AAAG)11 | FAM | 60 | 202 | 22 | 5 | 0.682 | 0.703 | 0.634 | 0.5665 |
| gpz-7 | F: TCCCATGTTGGGTGTAGTGA  R: AGCCACGTCTCATTTTCAAGA | KF907162 | (AAAG)11 | HEX | 63 | 216 | 21 | 10 | 0.714 | 0.822 | 0.779 | 0.1865 |
| gpz-8 | F: GGAGGGTACTTGCTGTGATGA  R: CTCCGACAAATTCGAGTACAGA | KF907163 | (AAAG)14 | FAM | 63 | 232 | 22 | 6 | 0.773 | 0.780 | 0.722 | 0.3419 |
| gpz-10 | F: ACACTTCCGTCTCTGGCTTC  R: CATCGTTGCAAACGGTAAGA | KF907164 | (AAAG)12 | HEX | 60 | 205 | 22 | 5 | 0.545 | 0.445 | 0.396 | 0.1304 |
| gpz-11 | F: GAGTCCGCTTCTCCCTCTG  R: TCAAATCCGCACAACACCTA | KF907165 | (AAAG)10 | TAMRA | 63 | 155 | 22 | 8 | 0.909 | 0.866 | 0.827 | 0.2321 |
| gpz-12 | F: CCTGCTTGTGCTCTCTCTAGC  R: AATTTGATCCATGGGTTGTTT | KF907166 | (AAAG)12 | FAM | 63 | 232 | 21 | 4 | 0.571 | 0.466 | 0.418 | 0.1139 |
| gpz-18 | F: AAAAATTCCATGTCCAAGAATGA  R: TCCTTAGTTGAGTATGCTTTCTGAG | KF907167 | (AAAG)13 | FAM | 60 | 209 | 22 | 7 | 0.864 | 0.740 | 0.682 | 0.0970 |
| gpz-19 | F: CCAAGGAAGTCCACATCCAG  R: TGCCTGCAAAGTTTCTGCTA | KF907168 | (AAAG)13 | TAMRA | 63 | 248 | 22 | 5 | 0.591 | 0.640 | 0.576 | 0.3257 |
| gpz-20 | F: CCCTCTCGTTGTGTCTCTCTG  R: CACCTGGTAAATGGCACCTT | KF907169 | (AAAG)10 | FAM | 63 | 248 | 20 | 8 | 0.800 | 0.759 | 0.714 | 0.2810 |
| gpz-25 | F: CCTGGTATCGAGTCCCACAC  R: TGACTGTTGGTGACTTGTCCTC | KF907170 | (AAAT)14 | HEX | 63 | 222 | 22 | 5 | 0.409 | 0.688 | 0.631 | 0.0055 |
| gpz-26 | F: CCTGTAAGGTGATGGTCTCCA  R: GGAAGGGGAAGAGAGAGACG | KF907171 | (AAAT)10 | TAMRA | 63 | 241 | 22 | 3 | 0.500 | 0.479 | 0.405 | 0.5124 |
| gpz-28 | F: AAGGGTTTGTTCCATTCACTG  R: CAACAGGCTAAACCGGTACAA | KF907172 | (AAAT)16 | HEX | 60 | 216 | 22 | 2 | 0.136 | 0.130 | 0.119 | 0.9308 |
| gpz-33 | F: TCATGATCTCCAGGTCCACA  R: TCAAAGGGTTTCCCATAGCA | KF907173 | (AAAT)12 | TAMRA | 60 | 235 | 22 | 3 | 0.182 | 0.286 | 0.262 | 0.9889 |
| gpz-36 | F: CCCCGTGTTAGGTTCTGTCT  R: GGAGTTGGCAACCTTTTGTT | KF907174 | (AAAT)12 | FAM | 60 | 168 | 22 | 2 | 0.591 | 0.460 | 0.348 | 0.1846 |
| gpz-40 | F: GCATTTCCTATGGCACCTGT  R: GCAGCATGCATTCATTTAGAGA | KF907175 | (AAAT)10 | HEX | 60 | 198 | 22 | 4 | 0.682 | 0.711 | 0.640 | 0.5085 |
| gpz-47 | F: GACCTCAGTGTACGCCCAGT  R: CTGGACAGGCAGGTAGAAGC | KF907176 | (AATG)20 | TAMRA | 60 | 230 | 22 | 4 | 0.545 | 0.532 | 0.479 | 0.5206 |
| gpz-48 | F: CCAGAGTTCTGGGATTGACC  R: CAGCTTGAAAGTTACCATTGGA | KF907177 | (AATG)11 | FAM | 60 | 211 | 22 | 2 | 0.091 | 0.089 | 0.083 | 0.9770 |
| gpz-50 | F: AAATGCCCCAACTCATTTCA  R: CTCCAGCCTGATATCCCAAA | KF907178 | (AATG)10 | HEX | 60 | 188 | 22 | 2 | 0.318 | 0.274 | 0.232 | 0.5699 |
| gpz-51 | F: GGGGAGGATATGTGTTGTGG  R: TGCTTTGGATTTATTGGAGCA | KF907179 | (AGAT)11 | TAMRA | 60 | 175 | 22 | 4 | 0.682 | 0.558 | 0.471 | 0.0587 |
| gpz-53 | F: CCGACTGCCTTATTTTGCAT  R: GAACAGCCCAAGTGTCCAAT | KF907180 | (AGAT)10 | FAM | 60 | 200 | 22 | 4 | 0.409 | 0.357 | 0.326 | 0.3617 |
| gpz-54 | F: CAATATTTTAAGGCGTGGGACT  R: GCATAATTGCAGAACCAGAGC | KF907181 | (AGAT)18 | TAMRA | 63 | 245 | 22 | 5 | 0.682 | 0.767 | 0.706 | 0.1978 |
| gpz-55 | F: TACATGCCCTCGGAGGTTTA  R: AGTTCCATTCATGTCATTTCAA | KF907182 | (AGAT)16 | HEX | 60 | 233 | 22 | 2 | 0.500 | 0.460 | 0.348 | 0.5267 |
| gpz-57 | F: CCATTTTGAATCCTGCCTTT  R: TCCAAAAATTGGAGACTATCCTG | KF907183 | (AGAT)12 | TAMRA | 60 | 230 | 22 | 4 | 0.682 | 0.608 | 0.538 | 0.2558 |
