# Supplementary material for: Genome-wide survey and analysis of microsatellites in giant panda (Ailuropoda melanoleuca), with a focus on the applications of a novel microsatellite marker system
Source: BMC Genomics. 2015 Feb 7;16(1):61. doi: 10.1186/s12864-015-1268-z (PMC4335702; doi:10.1186/s12864-015-1268-z)
Supplement: Additional file 4: Table S4. — The genotype result of the 13 captive giant panda faeces based on 6 microsatellites. [file 12864_2015_1268_MOESM4_ESM.doc]

Table S4. The genotype result of the 13 captive giant panda feces based on 6 microsatellites

|  | 6 microsatellites | | | | | |
| --- | --- | --- | --- | --- | --- | --- |
| Sample No. | GPL-29 | gpz-20 | gpz-06 | gpz-47 | GPL-60 | GPL-47 |
| M01 | 171/175 | 266/274 | 194/214 | 190/210 | 218/222 | 160/164 |
| M02 | 163/175 | 266/266 | 194/194 | 202/210 | 218/222 | 148/160 |
| M03 | 167/175 | 270/278 | 206/210 | 190/210 | 222/222 | 164/164 |
| M04 | 171/175 | 274/306 | 206/206 | 190/190 | 218/222 | 148/148 |
| M05 | 171/175 | 270/274 | 206/210 | 190/198 | 222/222 | 148/164 |
| M06 | 163/163 | 274/282 | 206/206 | 190/210 | 226/230 | 144/148 |
| M07 | 167/175 | 266/270 | 194/206 | 190/210 | 218/222 | 160/164 |
| M08 | 171/171 | 270/274 | 206/206 | 190/190 | 218/216 | 148/148 |
| M09 | 167/175 | 278/298 | 210/210 | 198/210 | 222/226 | 140/164 |
| M10 | 175/175 | 274/278 | 194/206 | 190/190 | 222/222 | 140/164 |
| M11 | 167/175 | 270/270 | 194/206 | 190/210 | 218/226 | 140/140 |
| M12 | 171/175 | 274/306 | 206/206 | 190/190 | 218/222 | 160/164 |
| M13 | 163/175 | 266/266 | 194/194 | 202/210 | 218/222 | 148/160 |
