# Supplementary material for: Genome-wide survey and analysis of microsatellites in giant panda (Ailuropoda melanoleuca), with a focus on the applications of a novel microsatellite marker system
Source: BMC Genomics. 2015 Feb 7;16(1):61. doi: 10.1186/s12864-015-1268-z (PMC4335702; doi:10.1186/s12864-015-1268-z)
Supplement: Additional file 5: Table S5. — The genotype result of the 22 unique wild giant panda individuals based on 6 microsatellites. [file 12864_2015_1268_MOESM5_ESM.doc]

Table S5. The genotype result of the 22 unique wild giant panda individuals based on 6 microsatellites

|  | GPL-29 | gpz-20 | gpz-06 | gpz-47 | GPL-60 | GPL-47 |
| --- | --- | --- | --- | --- | --- | --- |
| ww01 | 175/175 | 266/298 | 206/206 | 190/198 | 226/230 | 142/158 |
| ww03 | 163/175 | 274/298 | 206/206 | 190/198 | 226/230 | 142/166 |
| ww05 | 163/175 | 278/314 | 206/206 | 190/190 | 222/226 | 146/158 |
| ww08 | 171/175 | 270/278 | 194/206 | 190/190 | 222/230 | 146/146 |
| ww09 | 163/175 | 278/314 | 194/206 | 190/190 | 226/226 | 142/146 |
| ww10 | 163/167 | 274/274 | 194/206 | 190/198 | 226/234 | 0/0 |
| ww11 | 163/163 | 274/274 | 194/210 | 190/198 | 222/222 | 138/146 |
| ww12 | 163/175 | 0/0 | 210/210 | 190/202 | 226/238 | 158/162 |
| ww13 | 167/175 | 266/274 | 194/206 | 190/198 | 226/230 | 138/162 |
| ww14 | 171/175 | 274/326 | 194/202 | 210/210 | 226/226 | 142/158 |
| ww19 | 163/175 | 274/298 | 206/206 | 190/190 | 226/230 | 142/166 |
| ww20 | 167/175 | 266/274 | 194/206 | 190/198 | 226/230 | 138/162 |
| ww22 | 175/175 | 266/298 | 206/206 | 190/198 | 226/230 | 142/158 |
| ww29 | 163/171 | 274/274 | 194/206 | 190/210 | 226/226 | 142/162 |
| ww34 | 171/175 | 270/278 | 194/206 | 190/190 | 218/230 | 146/162 |
| ww36 | 163/175 | 0/0 | 206/210 | 190/210 | 218/222 | 138/162 |
| ww38 | 171/175 | 266/298 | 206/206 | 190/198 | 226/226 | 142/158 |
| ww48 | 163/163 | 274/278 | 206/206 | 190/190 | 218/222 | 158/162 |
| ww49 | 163/163 | 266/270 | 210/210 | 190/198 | 0/0 | 146/162 |
| ww52 | 163/167 | 278/278 | 198/206 | 198/198 | 218/226 | 138/138 |
| ww53 | 163/167 | 278/304 | 198/206 | 198/210 | 226/234 | 138/138 |
| ww59 | 163/175 | 266/266 | 198/202 | 190/190 | 226/238 | 162/162 |
